# Fractionated whole body γ-irradiation aggravates arthritic severity via boosting NLRP3 and RANKL expression in adjuvant-induced arthritis model: the mitigative potential of ebselen

**DOI:** 10.1007/s10787-023-01238-5

**Published:** 2023-05-02

**Authors:** Noura M. Thabet, Mohamed K. Abdel-Rafei, Mohamed M. Amin

**Affiliations:** 1 Radiation Biology Department, National Centre for Radiation Research and Technology (NCRRT), Egyptian Atomic Energy Authority, 3 Ahmed El-Zomor Street, Nasr City, P.O. Box 29, Cairo, 11787 Egypt; 2 Pharmacology Department, Medical Research and Clinical Studies Institute, National Research Centre, Dokki, Egypt

**Keywords:** NLRP-3, RANKL, Ebselen, γ-radiation, AIA, Collagen II

## Abstract

Rheumatoid arthritis (RA) is an autoimmune chronic inflammatory disease associated with oxidative stress that causes excruciating pain, discomfort, and joint destruction. Ebselen (EB), a synthesized versatile organo-selenium compound, protects cells from reactive oxygen species (ROS)-induced injury by mimicking glutathione peroxidase (GPx) action. This study aimed to investigate the antioxidant and anti-inflammatory effects of EB in an arthritic irradiated model. This goal was achieved by subjecting adjuvant-induced arthritis (AIA) rats to fractionated whole body γ-irradiation (2 Gy/fraction once per week for 3 consecutive weeks, for a total dose of 6 Gy) and treating them with EB (20 mg/kg/day, p.o) or methotrexate (MTX; 0.05 mg/kg; twice/week, i.p) as a reference anti-RA drug. The arthritic clinical signs, oxidative stress and antioxidant biomarkers, inflammatory response, expression of NOD-like receptor protein-3 (NLRP-3) inflammasome, receptor activator of nuclear factor κB ligand (RANKL), nuclear factor-κB (NF-κB), apoptotic indicators (caspase 1 and caspase 3), cartilage integrity marker (collagen-II), and histopathological examination of ankle joints were assessed. EB notably improved the severity of arthritic clinical signs, alleviated joint histopathological lesions, modulated oxidative stress and inflammation in serum and synovium, as well as reduced NLRP-3, RANKL, and caspase3 expression while boosting collagen-II expression in the ankle joints of arthritic and arthritic irradiated rats with comparable potency to MTX. Our findings suggest that EB, through its antioxidant and anti-inflammatory properties, has anti-arthritic and radioprotective properties in an arthritic irradiated model.

## Introduction

Rheumatoid arthritis (RA) is a chronic inflammatory disorder of connective tissue that causes joint lesions. As the disease progresses, patients experience intense pain, discomfort, and joint destruction, limiting their daily activities (Jin et al. 2018). This disease was caused due to an imbalance between autoimmunity and inflammation through dysregulation of pro- and anti-inflammatory cytokines production via macrophage and fibroblast (Kim et al. 2016). The NLRP-3 (NOD, LRR, and pyrin domain-containing protein-3) inflammasome, which is an essential component of the innate immune response and activated in RA, is among the pivotal pathogenesis keys in RA. Whereas it recognizes the pathogen-related or potentially dangerous signal molecules and activate caspase-1 (a pro-inflammatory protease). Activated caspase-1 cleaves the interleukin-1 β (IL-1β) precursors to produce the mature form, which induces secretion of Receptor activator of nuclear factor-κB ligand (RANKL) (Yu et al. 2021). Consequently, RANKL binds with its receptor RANK and induces activation of osteoclasts and bone resorption through stimulation of NF-κB (nuclear factor kappa-light-chain-enhancer of activated B cells), which increases pro-inflammatory cytokine secretion (Xu et al. 2012; Remuzgo-Martínez 2016). Through its regulation of various genes involved in inflammatory reactions, NF-κB is a crucial transcription factor. NF-κB translocates to the nucleus during inflammation and promotes the expression of inflammatory mediators that are essential in the development of RA, such as pro-inflammatory cytokines like tumor necrosis factor alpha (TNF-α), IL-1β, and IL-6 (Zhai et al. 2018). Moreover, it was found that reactive oxygen species (ROS) serve as an essential inflammasome activating signal (Harijith et al. 2014). Whereas it was shown that ROS are implicated in the pathophysiology of RA. Under normal conditions, ROS generation is controlled by a diversity of antioxidant defense systems, including non-enzymatic antioxidant defense (such as vitamins A and C, reduced glutathione; GSH) and enzymatic antioxidants (superoxide dismutase; SOD, catalase; CAT, and glutathione peroxidase; GPx) (Mateen et al. 2016). While oxidative stress is caused by an imbalance between ROS production and antioxidants as a result of amplified chemical reaction or a deficient antioxidant defense system, this condition causes joint damage due to highly reactive chemical species that have the potential to damage lipids, proteins, and DNA in joint tissues. As a result, if these ROS are not properly scavenged, they may cause damage to the biological macromolecules of the joint (Mateen et al. 2016). Furthermore, Watari et al. (2011) demonstrated that oxidative stress is related to arthritis development, most likely through the degradation of collagen-II in articular cartilage.

Exposure to environmental stressors such as ionizing radiation, toxic chemicals, and pollutants provokes oxidative stress accompanied by inflammation. Ionizing radiation, one of the exogenous environmental stressors, is threatening healthy and causes damage in tissues which eventually results in diseases. Workers in the nuclear power industry, researchers in radiological laboratories, and patients undergoing diagnostic procedures or routine therapeutic radiation are among those who are inevitably exposed to the deleterious effects of radiation (Azzam et al. 2012; Abdel-Rafei and Thabet 2020).

Methotrexate (MTX) is the most commonly used drug in RA therapy and other rheumatic diseases. The therapeutic action of MTX contributes to the suppression of inflammation in RA through inhibition of multiple pathways that stimulate sever bone inflammation, such as RANKL expression (Revu et al. 2010) and, subsequently, NF-κB-dependent pathways (Cronstein and Aune 2020). Ebselen (EB) (2-phenyl-1,2-benzisoselenazol-3(2H)-one) is one of the most relevant heterocyclic organo-selenium compounds that mimics GPx. Eb attenuates the H_2_O_2_ level in a mode similar to GPx, and also exhibits a broad range of biological activities, including antioxidant, cyto-protective properties, anti-atherosclerotic, anti-inflammatory, and anticancer activities (Thabet and Moustafa 2017; Abdel-Rafei et al. 2021).

The purpose of this study was to investigate the therapeutic potential of EB in an adjuvant- induced arthritis (AIA) while also exposing them to fractionated whole body γ-irradiation, relying on its bioactivity as anti-inflammatory and antioxidant. This goal was achieved through evaluating the expression of NLRP-3, RANKL, NF-κBp65 associated with the oxidative stress markers (ROS, malondialdehyde; MDA, GSH, SOD, and GPx), inflammatory mediators (TNF-α, IL-1β, IL-4 and IL-10), apoptotic indicators (caspase 1 and caspase 3), and cartilage integrity marker (collagen-II) and confirmed with histopathological examination of joint.

## Materials and methods

### Reagents and chemicals

Freund’s complete adjuvant (FCA) 1 mg of *Mycobacterium tuberculosis* (H37Ra, ATCC 25177), heat killed and dried, 0.85 mL paraffin oil and 0.15 mL mannide monooleate (CAT# F5881, Sigma-Aldrich). Ebselen (EB) was purchased from Sigma-Aldrich (St. Louis, MO, USA, CAT# 60940-34-3) as a powder (purity ≥ 98% TLC) was suspended in a 5% carboxymethyl cellulose (CMC) sterile saline solution (0.9% NaCl) (Otsuka Pharmaceuticals, Japan). Methotrxate (MTX) at a concentration of 25 mg/ml was acquired from EIMC United Pharmaceutics in Cairo, Egypt. The remaining chemicals and reagents were of high standard quality and analytical grade. The primary anti-RANKL antibody (Rabbit polyclonal antibody, Cell Signaling, CAT# 4816), rabbit polyclonal anti-NLRP3 antibody (Abcam, CAT# ab214185), rabbit polyclonal anti- Collagen II (Abcam, CAT# ab34712), and mouse monoclonal antibody against active Caspase-3 (anti-CASP3) (MyBiosource Inc., CAT # MBS9700318).

### Animals

The Wistar female albino adult rats (weighing 175–190 gm) used in this study were attained from the animal breeding unit of the National Center for Radiation Research and Technology (NCRRT) (Cairo, Egypt). Rats had free access to standard pellets and water ad libitum and were acclimatized for one week, at least before the beginning of experimental procedures.

### Ethics approval statement

Experimental rats were handled by following the recommendations of the National Institute of Health (NIH No 85:23, revised 1996) for the care and use of laboratory animals and in accordance with the regulations of Ethical Committee of the NCRRT, Atomic Energy Authority, Cairo, Egypt (Approval No. 35A/22).

### Adjuvant-induced arthritis (AIA) induction in rats

Adjuvant-induced arthritis (AIA) was developed in rats employing procedures proposed previously by Bao et al. (2019). Eight sham rats were chosen at random and received 0.1 mL of physiological saline subcutaneously into the plantar area of the footpad in the right hind paw just before induction. To induce AIA, rats received a single intradermal injection of 100 µL of dried and heat-killed (1 mg/mL) *M. tuberculosis* suspended in mineral oil (Freund's completed adjuvant; FCA). Shortly after the FCA injection, classic symptoms of inflammation were observed and culminated on day 12, with the day of FCA immunization being considered as day zero.

### Gamma irradiation facility and protocol

FCA-challenged AIA rats were exposed to a whole body γ-irradiation at a dose level of 2 Gy/fraction once per week for 3 consecutive weeks, for a total dose of 6 Gy, delivered at a dose rate of 0.401 Gy/min at the time of the experiment, following the guidelines of the Protection and Dosimetery Department, NCRRT. Rats were subjected to a whole body irradiation protocol, as previously demonstrated by Nylander et al. (2016) and Khalil and Al-Daoude (2019) utilizing total body irradiation approach with simultaneous pathological manifestations. Rats were irradiated at the NCRRT using Gamma Cell-40 biological irradiator with a Cesium-137 (Cs^137^) source (Atomic Energy of Canada Limited; Sheridan Science and Technology Park, Mississauga, Ontario, Canada). Rats were inserted into the Gamma cell-40 plastic sample tray that has exhaust vents that correspond to ventilation parts across the principle shield, to enable a process for uniform irradiation for small animals for every requisite irradiation exposure and kept for a sufficient amount of time to accomplish the exposure level.

### Experimental model

Rats were randomly grouped into 7 groups (eight rats/group) as follows: Group I (Sham group): normal rats just received a 5% CMC-Na solution vehicle. Group II (A group): rats were inoculated with 0.1 mL FCA and were orally gavaged with 5% CMC-Na solution. Group III (A + MTX group): FCA immunized rats were administered methotrxate (MTX), a reference anti-arthritic drug, at dose of (0.5 mg/kg; twice weekly, i.p) dissolved in physiological saline (Zhou et al. 2019). Group IV (A + EB group): AIA rats were given EB intragastrically at a dose of 20 mg/kg (Cheng et al. 2019), daily for two successive weeks. Group V (A + R group): AIA rats that were exposed to whole body γ-irradiation at a dose level of 2 Gy/fraction once per week for 3 consecutive weeks, up to a total dose of 6 Gy at a specific time points each week (middle of the week). Group VI (A + R + MTX group): AIA rats were exposed to fractionated whole body γ-irradiation and treated with MTX. Group VII (A + R + EB group): AIA rats were subjected to fractionated whole body γ-irradiation and treated with EB.

### Clinical evaluation of AIA severity

The severity of AIA was evaluated each three days post induction using body weight, paw swelling, polyarthritis index, and global arthritis assessment scores, as described earlier (Chang et al. 2016). At 3 day intervals, the body weights of the sham, A, and A + R groups, whether treated or untreated, were recorded. The body weights were recorded on day zero, before the FCA immunization. This was known as the initial body weight, although the value measured on other days has been known as the terminal body weight. The right hind paw volumes (mL) of every rat were estimated on day zero just before FCA immunization employing water displacement technique with a plethysmometer (UGO Basile, Italy) (Patil et al. 2012), and were observed each 3 days till the 21st day as primary swelling. The change in paw edema for each group is determined by subtracting the initial paw volume (basal) out from volume measured at each time point using following formula: (ml) = *V*_t_–*V*_0_, where V_0_ is the paw volume prior to FCA injection (ml) and *V*_t_ is the volume at (*t*) day after FCA immunization (ml) (Tong et al. 2018). The polyarthritis index scoring method spanned from 0 to 4 using a formerly reported macroscopic scoring technique (Bao et al. 2019), where 0 indicating no evidence of hyperemia; 1 denoting mild erythema and edema of ankle or wrist joints; 2 implying erythema and swelling of paws; 3 inferring severe inflammation of the whole paw; and 4 demonstrating entire limb and digits with substantial swelling and malformation. The polyarthritis index score was determined by adding the cumulative scores from each rat's four paws, with a peak value of 16. The global arthritis assessment score is obtained using macroscopic examination of clinical symptoms in multiple aspects of AIA rats, where 0 = no nodule and erythema; 1 = nodule and redness in one ear; 2 = nodule and redness across both ears, Nose: 0 if there is no connective tissue swelling and erythema; 1 if there is visible connective tissue swelling and erythema. Tail: 0 if there is no nodule and no redness, 1 if there is a noticeable nodule and erythema. Paws: 0 if there is no swelling and redness, 1 denoting one paw with swelling, 2 inferring mild swelling and erythema in two paws, 3 implying severe swelling and erythema in three paws, and 4 indicating redness, intense swelling, and malformation in four paws (Xiu et al. 2015).

### Sampling and preparation of blood and tissue

The experimental procedures started after the development of arthritis on day 9, then exposed to radiation on day 12 and certain time points in each week successively. Under gentle anesthesia, 1 h immediately following the last drug dosing, blood samples were gathered by cardiac perforation, permitted to coagulate at ambient temperature, and then centrifuged at 4000 rpm for 15 min using a centrifuge (Hettich Universal 32A, Germany). The collected sera was further separated and kept at − 80 °C until use. Ankle joins and synovial tissues were excised. A portion was then washed in ice-cold saline solution and homogenates were prepared for biochemical estimation, while the remaining part was used for histopathological investigation. The total protein content in the tissue aliquots was determined using the Bradford method (Bradford 1976).

### Histopathological examination of tibiotarsal joints

The ankle joints were excised out after sacrifice on the last day of the experiment and promptly fixed in 4% paraformaldehyde and decalcified in 10% ethylenediaminetetraacetic acid (EDTA) for 30 days till the complete decalcification at 4 °C. Tissue samples were cleared up in xylene and enclosed in paraffin for 24 h at 56 degrees in a hot air oven. To prepare paraffin beeswax tissue blocks for slicing at 5 µm thicknesses, a sledge microtome was used. The tissue sections were obtained, deparaffinized, and stained with hematoxylin and eosin (H&E) stain for examination under a light electric microscope (Bancroft et al. 1996). Tissue sections were graded for inflammatory cell infiltration, synovial proliferation, pannus formation, and cartilage damage on a scale of 0–3 (0 = none, 1 = mild, 2 = moderate, and 3 = severe), and the average score was calculated (Wei et al. 2013; Xiang et al. 2016). An unbiased pathologist who was unaware of the therapeutic regimen accomplished the distinctive pathological debilitating lesions of the tibiotarsal joints blindly and at random using an Olympus CX41 light microscope (Olympus, Tokyo, Japan) equipped with a high resolution digital camera system.

### Biochemical assays

The level of reactive oxygen species (ROS) was quantified using a commercially available rats’ enzyme linked immunosorbent (ELISA) kit purchased from MyBioSource (San Diego, CA, USA; CAT# MBS039665). Lipid peroxidation, in terms of malondialdehyde (MDA), was estimated according to the method of Yoshioka et al. (1979), and the reduced glutathione (GSH) content was measured as described by Ellman (1959). The activity of glutathione peroxidise (GSH-Px) was determined according to the method of Gross et al. (1967). The antioxidant enzymes, superoxide dismutase **(**SOD) and catalase (CAT) activities were assayed by the methods of Kakkar et al. (1984) and Bergmeyer et al. (1988), respectively. The levels of pro- and anti-inflammatory mediators such as IL-1β, TNF-α, IL-4, and IL-10 were measured using the corresponding specific rat ELISA kits from Abcam (Cambridge, UK; CAT# ab100768, CAT# ab100785, CAT# ab100770, and CAT# ab214566, respectively), while the rat caspase-1 ELISA kit from MyBioSource (CAT# MBS765838) was utilized to estimate caspase-1 level. These biochemical indices were measured in the serum and synovial tissues of arthritic and arthritic irradiated rats, whether treated or untreated, according to the manufacturers’ instructions. For the detection of NF-κB p65 in synovial tissue, synovial specimens were sliced into small pieces and incubated for 24 h in 2 ml of serum-free RPMI-1640 (Thermo Fischer Scientific, Rockford, IL, USA) containing 0.25% lactalbumin hydrolysate in a 5% CO2 incubator.. Tissue samples were homogenized on ice in a 50 mM Tris–HCl buffer with a pH of 7.5 (Chang et al. 2015). Tissue homogenates were used to prepare nuclear extracts as previously illustrated (Lee et al. 2006). In the yielded nuclear extracts, the level of NF-κB p65 was assessed using an ELISA kit (MyBiosource; CAT# MBS015549) as per the manufacturers’ instructions.

### Immunostaining of RANKL, NLRP3, collagen-II, and caspase-3 in ankle joints

Immunohistochemical detection of RANKL, NLRP3, Collagen II, and Caspase-3 expression was performed on 4 µm thick demineralized ankle joint sections. All sections were dewaxed by xylene, dehydrated, and thoroughly washed three times in PBS for 5 min before being incubated with 3% H_2_O_2_ for ten min at 37 °C. They were then rinsed 3 times in PBS before being blocked with goat serum blocking solution. Excess fluid was removed, diluted primary anti-RANKL (1:50, Cell Signaling, CAT# 4816), anti-NLRP3 (1:300, Abcam, CAT# ab214185), anti-Collagen II (1:200, Abcam, CAT# ab34712), and anti-Caspase-3 (1:100, MyBiosource, CAT # MBS9700318) were placed drop by drop, and sections were preserved in a humidified cabinet at 4 °C for 24 h. After 20 min of stability and shaking at 37 °C, the sections were washed thoroughly and incubated for 20 min with a streptavidin–horseradish peroxidase-labeled secondary antibody. The yellow 3, 3′-diaminobenzidine (DAB) staining and hematoxylin counterstaining were used to view the expression of biomarkers. Phosphate-buffered saline (PBS) served as a negative control. To ascertain immunostaining, immunohistochemical analysis was carried out using Image-Pro Plus 6.0 software.

### Statistical analysis

Statistical analysis was accomplished by one-way analysis of variance (ANOVA) then followed by Tukey–Kramer multiple comparison tests, except for analysis of changes in body weight, arthritis score, edema volume, and global polyarthritis assessment which used two-way ANOVA followed by the post hoc Dunnette’s test for multiple comparisons. The scored data of selected histopathological parameters were presented as median and range, and the difference between all tested groups was analyzed using Mann–Whitney *U* test for non-parametric analysis. The Kolmogorov–Smirnov (KS, *P* > 0.10) test was used to confirm data normality, and the proper test was used when needed. Graph Pad prism 8 was used for statistical analysis (Graph Pad Software Inc, San Diego, California, USA). Data were expressed as mean values ± standard error of the mean (SEM) and differences between values are considered significance at *P* ˂ 0.05.

## Results

### EB alleviates clinical arthritic indices in arthritic-irradiated rats

**Fig. 1:**
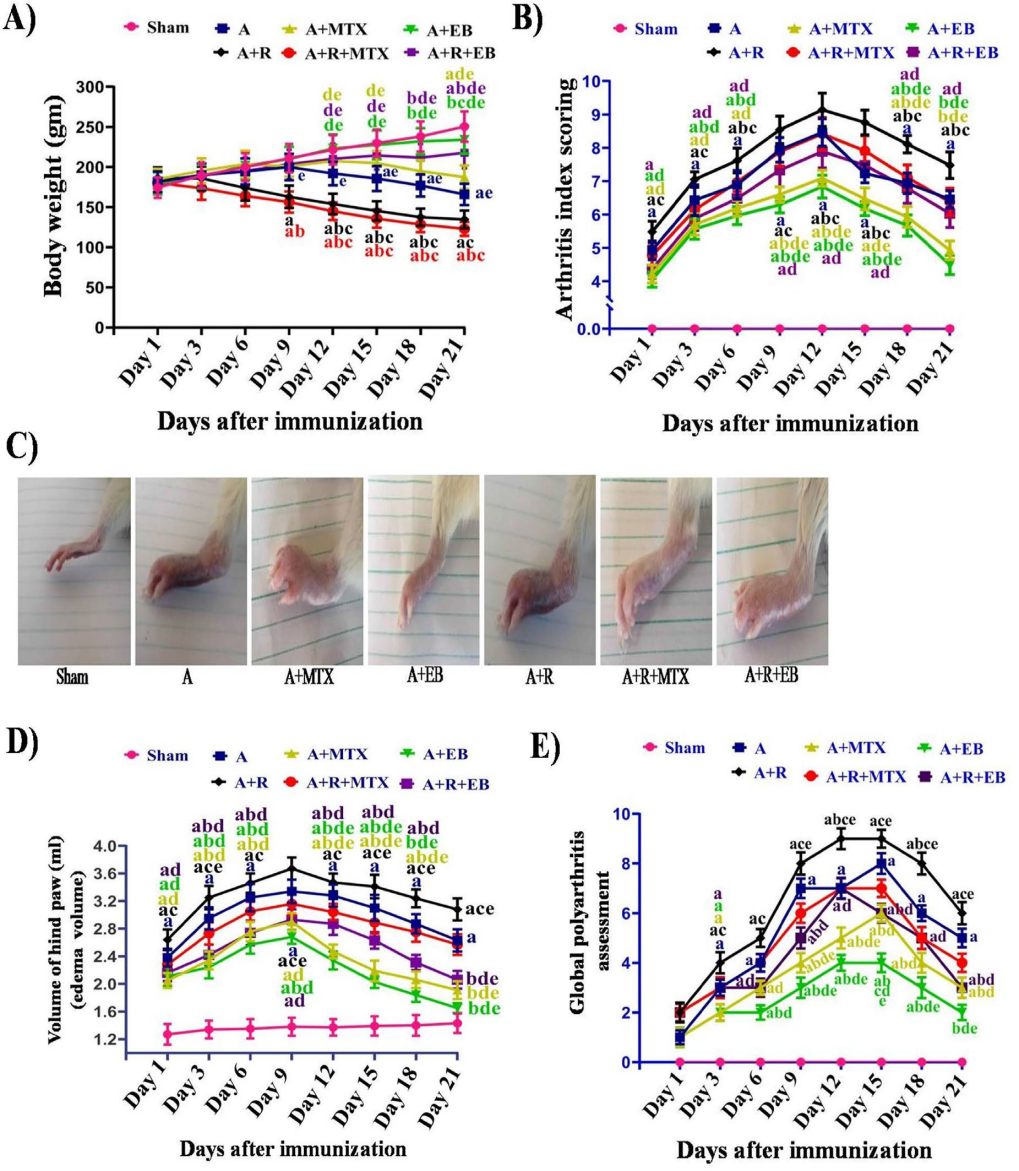
Impact of EB on the severity and progression of arthritic clinical signs in arthritic-irradiated rats. **A** Differences in body weight gain in sham and arthritic rats were recorded every 3 days post FCA inoculation till the end of experiment (day 21). **B** Arthritic polyarthritis index score from day 1 to day 21 following FCA challenge. **C** Representative images of the swollen hind paws in each group. **D** The difference in right hind paw circumference (paw swelling) in the A model group and irradiated arthritic rats compared to AIA treated and Sham groups recorded each 3 days after FCA immunization. **E** The global polyarthritis assessment scoring in all groups recorded from day 1 to day 21 after FCA immunization. Each value represents Mean ± SEM (*n* = 6–8). Columns denoted with ״a״ significant from sham, ״b״: significant from A, ״c״ significant from A + MTX, ״d״ significant from A + R, and ״e״ significant from A + R + MTX, at (*P* < 0.05). These colored significant letters correspond to the respective group. Sham: normal rats, A: rats injected with FCA, A + MTX: rats injected with FCA and treated with MTX, A + EB: rats injected with FCA and treated with EB, A + R: rats injected with FCA and exposed to 2 Gy/week; up to a cumulative dose of 6 Gy, A + R + MTX: rats injected with FCA, and exposed to2 Gy/week; up to a cumulative dose of 6 Gy and treated with MTX, and A + R + EB: rats injected with FCA, and exposed to 2 Gy/week; up to a cumulative dose of 6 Gy and treated with EB (color figure online)

Every 3 days after AIA induction, rats' body weight gain, ankle circumferences, and inflammatory clinical arthritic scores, including paw volume, arthritis index score, and global polyarthritis assessment, were measured (Fig. 1A–E). Body weight gain in animals is widely regarded as a reliable predictor of the anti-inflammatory and immunomodulatory outcomes of the medications assessed in the AIA model. Over the first six days after FCA immunization, the average body weight of rats in sham, A, A + MTX, and A + EB groups increased (Fig. 1A). In the A + R and A + R + MTX groups, a steady decline in mean body weight of rats begins within the initial three days after FCA challenge. While, as secondary arthritis progressed, the mean body weights of rats immunized with FCA dropped drastically as revealed in A group. Conversely, the average body weights of rats in the sham group rose over time. Notably, the A + R and A + R + MTX groups demonstrated a significant reduction (*P* ˂ 0.05) in mean body weight beginning on day 9 and clearly diminished continuously to the last day of experiment when compared to sham, A, A + MTX, A + EB, and A + R + EB groups. However, in the A + MTX group, a noticeable reduction (*P* ˂ 0.05) in mean body weight was only observed on day 15 and peaked at day 21. Oral administration of EB at a dose of 20 mg/kg daily to either arthritic or arthritic irradiated rats (A + EB and A + R + EB groups) efficiently maintained the mean body weight of rats across all the stages of arthritis progression (primary and secondary arthritis) as compared to the A, A + MTX, A + R, and A + R + MTX groups (Fig. 1A). In Fig. 1B, the mean arthritic index score for the A and A + R groups implies that secondary arthritis has started on day 9 and increased substantially by day 12 when compared to the sham set, with the highest score recorded in the A + R group as compared to the respective A group. A significant improvement (*P* ˂ 0.05) was observed in the mean arthritis index score of the A + MTX group when compared to the A, A + R, and A + R + MTX groups (Fig. 1B). Intriguingly, the A + EB group exhibited a mean arthritic index score profoundly lower (*P* ˂ 0.05) than those recorded in the corresponding A, A + R, and A + R + MTX groups. On the other hand, in the A + R + EB group, treatment of arthritic irradiated rats with EB effectively reduced (*P* ˂ 0.05) the mean arthritis index score as compared to the A + R group, with a comparable score to that recorded in the A + R + MTX group, but with a better improvement. Macroscopic photographs and measurement of paw edema from FCA-inoculated hind paws were employed to evaluate the degree of inflammation among groups (Fig. 1C, D). An acute inflammatory and autoimmune phase of swelling was induced after day 6 of FCA inoculation in both inoculated and non-inoculated paws that surged at day 9 (*P* ˂ 0.05, 143%), and this increase in paw circumference was prolonged to day 21, with a steady decrement to 86% on the last day of experiment (day 21) in the A group compared to the sham rats (Fig. 1D). Whereas, in the A + R group, a remarkable rise (*P* ˂ 0.05, 168%) in paw volume started on day 9 after the FCA challenge and continued with an gradual lowering to reach 117% and 18% when compared to the sham and A groups, respectively, on day 21. When administered twicely/week at a dose of 0.05 mg/kg, MTX exerted a significant abolishment (*P* ˂ 0.05) in paw volume by 28% in A + MTX group as compared to A group and by 18% in the A + R + MTX group as compared to A + R group on day 21. Most importantly, the A + EB and A + R + EB groups showed a pronounced improvement (*P* ˂ 0.05) in paw swelling as perceived by a decrease in paw volume of 38% in the A + EB group as compared to the A group and by 32% in the A + R + EB group when compared to the A + R group on day 21 of the experimental course (Fig. 1D). As compared to the sham group, rats in the A and A + R groups had systemic inflammation and the evolution of arthritis was transient, as indicated by the emergence of nodules on the tail and non-inoculated paws, as well as redness of the ears and nose, with the utmost global polyarthritis assessment score (*P* ˂ 0.05) at days 12 and 15 following FCA immunization (Fig. 1E). The A + MTX, A + EB, A + R + MTX, and A + R + EB groups exhibited a profound improvement in their global polyarthritis assessment scores when compared to the A and A + R groups, with the most improvement observed in the A + EB and A + R + EB groups when compared to the A + MTX and A + R + MTX groups, respectively (Fig. 1E).

### EB ameliorates degenerative changes in ankle joints of arthritic-irradiated rats

**Fig. 2:**
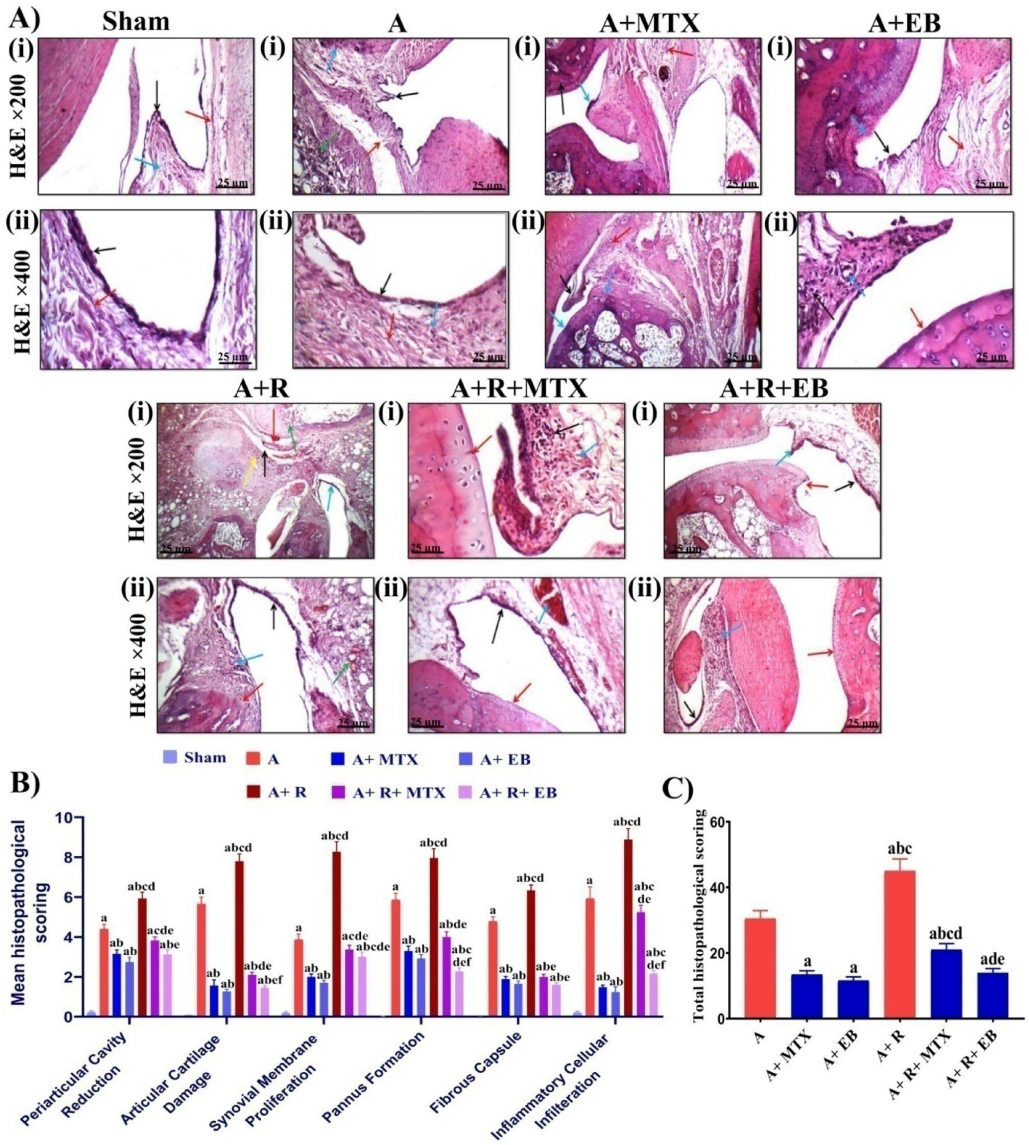
EB attenuates pathological lesions in ankle joints and cartilages of arthritic-irradiated rats. **A** Representative photomicrographs of H&E stained tibiotarsal joints of sham and arthritic rats (magnification X 200 & X 400) for all groups. **B** Quantification of individual histopathological lesion score. **C** Assessment of the total quantified histopathological lesions scoring of joints was determined and the overall mean was calculated in each group. Each value represents Mean ± SEM (*n* = 6–8). Mann–Whitney *U* test for non-parametric analysis was used to indicate the difference between all tested groups to compare the specific individual histopathological score. Columns denoted with ״a״ significant from sham, ״b״: significant from A, ״c״ significant from A + MTX, ״d״ significant from A + EB, ״e״ significant from A + R and ״f״ significant from A + R + MTX, at (*P* < 0.05). Sham: normal rats, A: rats injected with FCA, A + MTX: rats injected with FCA and treated with MTX, A + EB: rats injected with FCA and treated with EB, A + R: rats injected with FCA and exposed to 2 Gy/week; up to a cumulative dose of 6 Gy, A + R + MTX: rats injected with FCA, and exposed to2 Gy/week; up to a cumulative dose of 6 Gy and treated with MTX, and A + R + EB: rats injected with FCA, and exposed to 2 Gy/week; up to a cumulative dose of 6 Gy and treated with EB (color figure online)

As discerned in Fig. 2A, we then examined the ability of EB to mitigate the extent of injury in the tibiotarsal joints of AIA rats' ankles using histopathological analysis. The joint architecture in H&E stained sections of sham rats shows an average joint cavity with an intact normocellular synovial membrane (black arrow-i), average fibrous capsule (blue arrow-i), and an average surrounding soft tissue (red arrow-i) (H&E X 200). An intact normocellular superficial layer of synovial membrane (black arrow-ii) and average surrounding soft tissue (red arrow-ii) are visible at a high magnification power (H&E X 400). Contrarily, moderate degenerative lesions were observed in the examined joint sections of the A group as represented by pannus formation (black arrow-i), destructing articular cartilage (blue arrow-i), destructed fibrous capsule (red arrow-i), and moderate inflammatory infiltrate in the surrounding soft tissue (green arrow-i) (H&E X 200). In addition, an intact synovial membrane (black arrow-ii), with a large pannus showing a moderated inflammatory infiltrate composed mainly of lymphocytes (blue arrow-ii) and fibroblasts (red arrow-ii), was revealed on high power view (H&E X 400). Meanwhile, an improvement was perceived in the assessed joint sections of the A + MTX group as indicated by a focally hyperplastic synovial membrane (black arrow-i), intact normocellular articular cartilage (blue arrow-i), and a mild inflammatory infiltrate in the surrounding soft tissue (red arrow-i) (H&E X 200). Another view elucidating the narrow joint cavity with pannus formation (black arrow-ii) and intact underlying articular cartilage (blue arrow-ii), and mild inflammatory infiltrate in surrounding soft tissue (red arrow-ii) (H&E X 400). Along with that, but to a greater extent, the scrutinized joint sections of the A + EB group displayed minor joint damage as manifested by the formation of tiny pannus with underlying mild inflammatory infiltrate (black arrow-i), focally destructed articular cartilage (blue arrow-i), and mild edema in surrounding soft tissue (red arrow-i) (H&E X 200). Moreover, at high power magnification, a small pannus with an underlying mild inflammatory infiltrate composed mainly of lymphocytes (black arrow-ii) and thick-walled blood vessels (blue arrow), as well as intact normocellular articular cartilage (red arrow-ii) was noticed (H&E X 400). In A + R group examined sections, arthritic rats were exposed to fractionated whole body γ-irradiation, which exacerbated the severity of the joint pathological lesions as implied by the narrow joint cavity (black arrow-i) with diffuse synovial hyperplasia (blue arrow-i), accompanied by small pannus formation (red arrow-i), focally destructed articular cartilage (green arrow-i), and the surrounding soft tissue showing marked inflammatory infiltrate (yellow arrow-i) (H&E X 200). Another view showing diffuse synovial hyperplasia (black arrow-ii) with large pannus formation (blue arrow-ii), destructed articular cartilage (red arrow-ii), and a marked inflammatory infiltrate with congested blood vessels in surrounding soft tissue (green arrow-ii) (H&E X 400). Whereas, in the A + R + MTX group, the examined joint sections showed a reasonable improvement in the preceding lesions, as evidenced by an average articular cartilage (red arrow-i) with large pannus formation, as well as a marked inflammatory infiltrate composed primarily of lymphocytes (black arrow-i), and mildly congested blood vessels (blue arrow-i) (H&E X 200). A further view depicts diffuse hyperplastic synovial membrane (black arrow-ii), large pannus with markedly congested blood vessels (blue arrow-ii), and intact normocellular articular cartilage (red arrow-ii) (H&E X 400). Interestingly, a promising amelioration was observed in the examined joint sections from the A + R + EB group as represented by a focally destructed normocellular articular cartilage (red arrow-i) and diffuse synovial hyperplasia (black arrow-i) with a small pannus showing mild inflammatory infiltrate (blue arrow-i) (H&E X 200). Also, a high power view showing diffuse synovial hyperplasia (black arrow-i) with a small pannus showing moderated inflammatory infiltrate (blue arrow-ii), and intact normocellular articular cartilage (red arrow-ii) (H&E X 400). A summary of the quantified histopathological lesion scoring to discriminate the severity between groups is illustrated in Fig. 2B and C. Obviously, when compared to the sham set, a significant increase in the pathological lesion scoring was revealed in the A, A + R, A + R + MTX, A + R + EB, A + MTX, and A + EB groups, while these degenerative changes were attenuated considerably in the A + EB, A + MTX, A + R + EB, and A + R + MTX groups, as compared to the A and A + R groups (Fig. 2B and C).

### EB abates oxidative stress evoked in arthritic-irradiated model

**Fig. 3:**
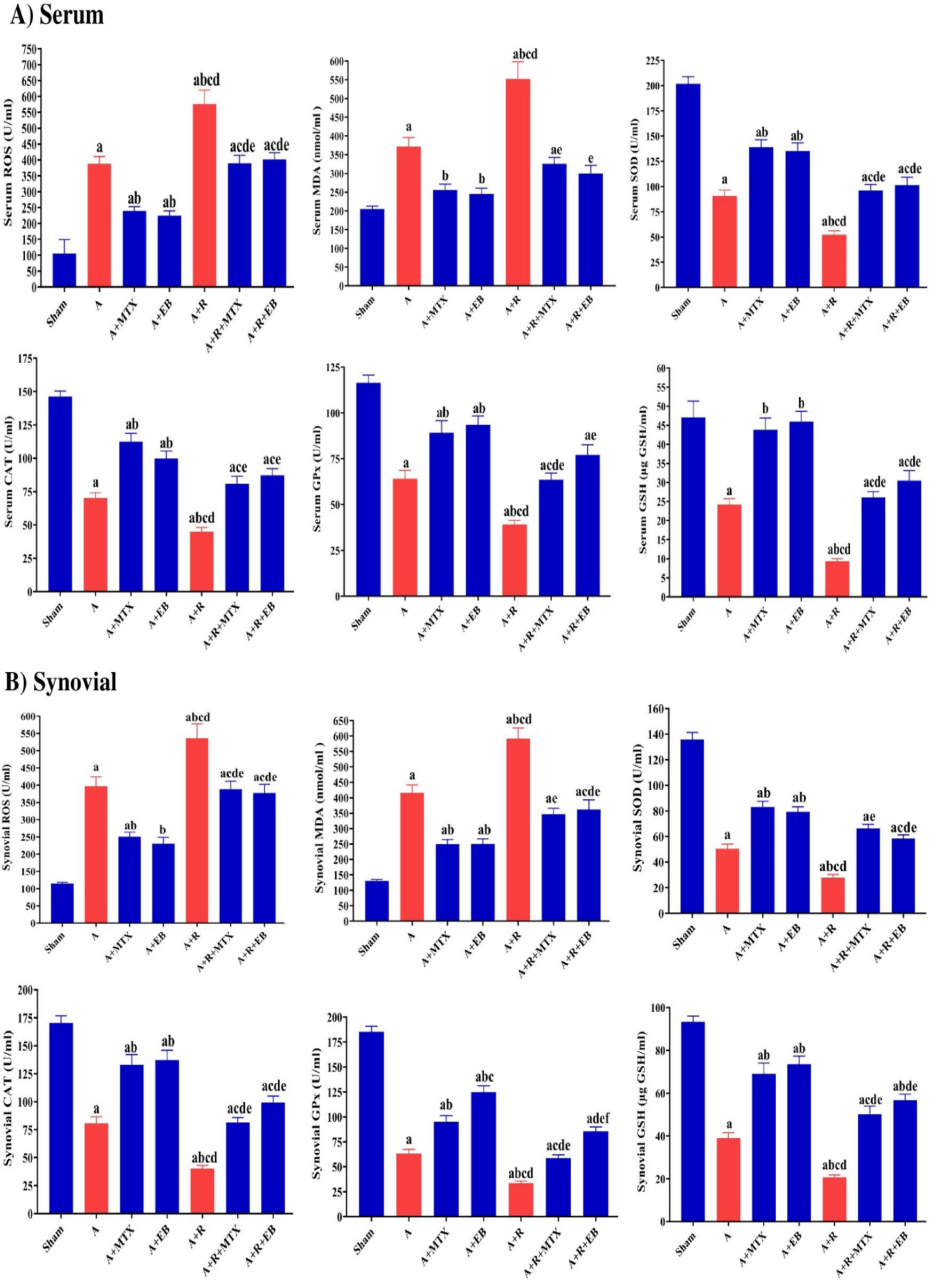
Antioxidant capacity of EB mediated through combating oxidative stress and replenishing the cyto-protective machinery in arthritic-irradiated rats. **A** Serum levels of ROS and MDA as well as SOD, CAT, and GPx activities, besides GSH content. **B** Synovial levels of ROS and MDA as well as SOD, CAT, and GPx activities, besides GSH content. Each value represents Mean ± SEM (*n* = 6–8). Columns denoted with ״a״ significant from Sham, ״b״: significant from A, ״c״ significant from A + MTX, ״d״ significant from A + EB, ״e״ significant from A + R and ״f״ significant from A + R + MTX, at (*P* < 0.05). Sham: normal rats, A: rats injected with CFA, A + MTX: rats injected with CFA and treated with MTX, A + EB: rats injected with CFA and treated with EB, A + R: rats injected with CFA and exposed to 2 Gy/week; up to a cumulative dose of 6 Gy, A + R + MTX: rats injected with CFA, and exposed to2 Gy/week; up to a cumulative dose of 6 Gy and treated with MTX, and A + R + EB: rats injected with CFA, and exposed to 2 Gy/week; up to a cumulative dose of 6 Gy and treated with EB (color figure online)

When compared to the sham group, the data from the A group showed a significant upregulation (*P* ˂ 0.05) of ROS levels in serum by 3.7-folds and in synovial tissue by 3.5-folds, accompanied by a surge (*P* ˂ 0.05) of MDA levels in serum by 1.81-folds and in synovial tissue by 3.2-folds, as shown in Fig. 3A and B. These results were associated with a substantial decline (*P* ˂ 0.05) in the antioxidant defensive machinery of the A group. This was represented by a considerable decrement in SOD, CAT, and GPx activities as well as GSH levels in serum by 54.99%, 51.99%, 44.96%, and 48.62%, respectively, and in synovial tissue by 62.95%, 52.70%, 65.88%, and 58.17%, respectively, as compared to the respective sham set.

The data of the A + MTX group revealed a significant improvement (*P* ˂ 0.05) against arthritic status through diminishing ROS (by 38.36% and 36.87%) and MDA (by 31.13% and 39.95%) levels, along with a significant augmentation (*P* ˂ 0.05) in SOD (by 1.53- folds and 1.65-folds), CAT (by 1.60-folds and 1.65-folds), GPx (by 1.4-folds and 1.51-folds) activities as well as GSH level (by 1.81-folds and 1.8- folds) in serum and synovial tissue, respectively, when compared with the A group (Fig. 3A and B).

The administration of EB at a dose of 20 mg/kg daily to arthritic rats as perceived in the A + EB group efficiently counteracted (*P* ˂ 0.05) oxidative stress through decreasing levels of ROS (by 42% and 37.39%) and MDA (by 34.11% and 39.78%), coupled with boosted (*P* ˂ 0.05) enzymatic antioxidant biomarkers including SOD (by 1.5-folds and 1.6-folds), CAT (by 1.42-folds and 1.70-folds), GPx (by 1.46-folds and 2- folds) activities and GSH level (by 1.90-folds and 1.88-folds) in serum and synovial tissue, respectively, as compared to the A group (Fig. 3A and B).

When compared to the A group, exposure of arthritic rats to fractionated whole body γ-irradiation as shown in the A + R group resulted in a significant rise (*P* ˂ 0.05) of ROS and MDA levels in serum by 1.5-folds and 1.5-folds and synovial tissue by 1.35-folds and 1.45-folds, respectively. This elevation in oxidative stress indicators was correlated with a pronounced curtailment (*P* ˂ 0.05) in antioxidant markers of the A + R group, as implied by a noticeable decrement in SOD, CAT, and GPx activities as well as GSH level in serum by 42.17%, 36.05%, 39.05% and 61.33%, respectively, and in synovial tissue by 44.61%, 49.93%, 46.39% and 47.08%, respectively, as compared to the A group (Fig. 3A and B).

In the A + R + MTX group, the obtained data demonstrated a significant modulation (*P* ˂ 0.05) in the oxidative stress status through a decrease in the levels of ROS by 32.36% and 27.42% and MDA by 41.03% and 41.32%, paralleled by a significant reinforce (*P* ˂ 0.05) in the activities of SOD (by 1.83- folds and 2.4- folds), CAT (by 1.80 folds and 2.02-folds), GPx (by 1.63-folds and 1.73-folds) and level of GSH (by 2.8-folds and 2.4-folds) in serum and synovial tissue, respectively, when compared to the A + R group, as displayed in Fig. 3A and B.

After EB supplementation to the arthritic irradiated rats, as represented by the A + R + EB group, the results exhibited a remarkable protection (*P* ˂ 0.05) against oxidative stress status portrayed in the A + R group through restraining the levels of ROS (by 30.35% and 29.64%) and MDA (by 45.63% and 38.87%) concomitantly with a significant upregulation (*P* ˂ 0.05) in antioxidant system via augmenting the activities of SOD (by 1.93-folds and 2.1-folds), CAT (by 1.94-folds and 2.5-folds), and GPx (by 2-folds and 2.53-folds) activities as well as the level of GSH (by 3.26-folds and 2.8-folds) in serum and synovial tissue, respectively, as compared to the A + R group (Fig. 3A and B).

### EB improves inflammatory response in arthritic-irradiated rats

**Fig. 4:**
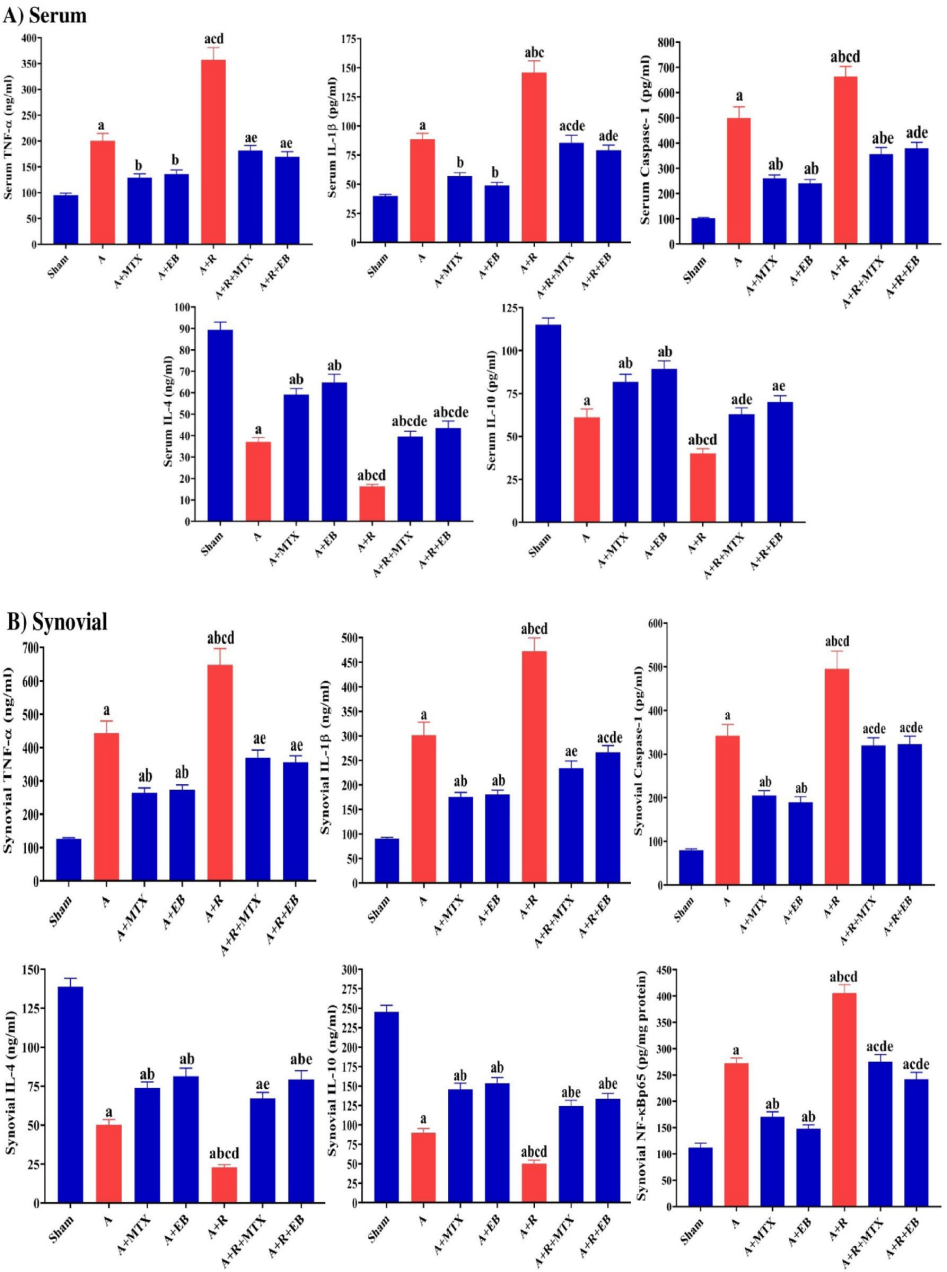
Modulatory effect of EB on the pro- and anti-inflammatory mediators in arthritic-irradiated rats. **A** Serum TNF-α, IL-1β, caspase-1, IL-4, and IL-10 levels. **B** Synovial TNF-α, IL-1β, caspase-1, IL-4, IL-10, and NF-κBp65 levels. Each value represents Mean ± SEM (*n* = 6–8). Columns denoted with ״a״ significant from Sham, ״b״: significant from A, ״c״ significant from A + MTX, ״d״ significant from A + EB, ״e״ significant from A + R and ״f״ significant from A + R + MTX, at (*P* < 0.05). Sham: normal rats, A: rats injected with CFA, A + MTX: rats injected with CFA and treated with MTX, A + EB: rats injected with CFA and treated with EB, A + R: rats injected with CFA and exposed to2 Gy/week; up to a cumulative dose of 6 Gy, A + R + MTX: rats injected with CFA, and exposed to 2 Gy/week; up to a cumulative dose of 6 Gy and treated with MTX, and A + R + EB: rats injected with CFA, and exposed to 2 Gy/week; up to a cumulative dose of 6 Gy and treated with EB (color figure online)

Figure 4A and B depicts the inflammatory status biomarkers, which include both pro-inflammatory mediators (TNF-α and IL-1β) as well as the anti-inflammatory cytokines (IL-4 and IL-10) concurrently with caspase-1. When compared with the sham group, the data of the A group showed a marked rise (*P* ˂ 0.05) in the levels of TNF-α (by 2.2-folds and 3.52-folds), IL-1β (by 2.21-folds and 3.35-folds), and caspase-1 (by 4.87- folds and 4.3-folds) concordantly with a noteworthy diminution (*P* ˂ 0.05) in the levels of IL-4 (by 58.52% and 63.87%) and IL-10 (by 46.98% and 63.26%) in serum and synovial tissue, respectively.

When compared to the A group, the A + MTX group had a significant alleviation (*P* ˂ 0.05) in the inflammatory status, with lower levels of TNF-α (by 35.46% and 40.42%), IL-1β (by 35.65% and 41.78%), and caspase-1 (by 47.83% and 39.89%), which was correlated with enhanced levels of IL-4 (by 1.6-folds and 1.5-folds) and IL-10 (by1.35-folds and 1.7-folds) in serum and synovial tissue, respectively (Fig. 4A and B).

The data presented in Fig. 4 A and B revealed a significant modulation (*P* ˂ 0.05) in inflammatory markers in the A + EB group, including a notable decrease in the levels of TNF-α (32.32% and 38.62%), IL-1β (44.74% and 40.28%), and caspase-1 (51.82% and 44.65%), as well as an elevation in the levels of IL-4 (by 1.75-folds and 1.65-folds) and IL-10 (by 1.46-folds and 1.71-folds) in serum and synovial tissue, respectively, when compared to the A group.

Exposure of arthritic rats to fractionated whole body γ-radiation (A + R group) markedly raised (*P* ˂ 0.05) the levels of TNF-α (by 1.78-folds and 1.5-folds), IL-1β (1.64-folds and 1.6-folds), and caspase-1 (by 1.33-folds and 1.5-folds), accompanied by a significant diminish (*P* ˂ 0.05) in the levels of IL-4 (by 55.92% and 54.02%) and IL-10 (by 34.30% and 44.12%) in serum and synovial tissue, respectively, when compared to the A group (Fig. 4A and B).

As demonstrated in the A + R + MTX group, MTX treatment of arthritic irradiated rats resulted in a significant modulation (*P* ˂ 0.05) in inflammatory status via reductions in the levels of TNF-α (by 49.22% and 43.05%), IL-1β (by 41.28% and 50.49%), and caspase-1 (by 46.33% and 28.48%), with an augment in the levels of IL-4 (by 2.42-folds and 3-folds) and IL-10 (by 1.57-folds and 2.5-folds) in serum and synovial tissue, respectively, as compared to the A + R group (Fig. 4A and B).

When compared with the A + R group, the A + R + EB group exhibited profound amelioration (*P* ˂ 0.05) in inflammatory markers via a decrease in the levels of TNF-α (by 52.55% and 45.12%), IL-1β (by 45.76% and 43.58%), and caspase-1 (by 42.69% and 34.92%), as well as an increase in the levels of IL-4 (by 2.66-folds and 3.45-folds) and IL-10 (by 1.75-folds and 2.65-folds) in serum and synovial tissue, respectively, as shown in Fig. 4A and B.

The synovial NF-κBp65 level showed a marked elevation (*P* ˂ 0.05) in the A and A + R by 2.5-folds and 3.65-folds, respectively, as compared to the sham rats (Fig. 4B). When compared to the A group, a significant reduction (*P* ˂ 0.05) in synovial NF-κBp65 level by 37.5% and 45% was shown in the A + MTX and A + EB groups, respectively. The treatment of arthritic irradiated rats with MTX and EB produced a noticeable curtailment (*P* ˂ 0.05) in synovial NF-κBp65 level by 33% and 40%, as represented by the A + R + MTX and A + R + EB groups, respectively, when compared to the A + R group (Fig. 4B).

### EB downregulates RANKL and NLRP3 expression in joints of arthritic-irradiated rats

**Fig. 5:**
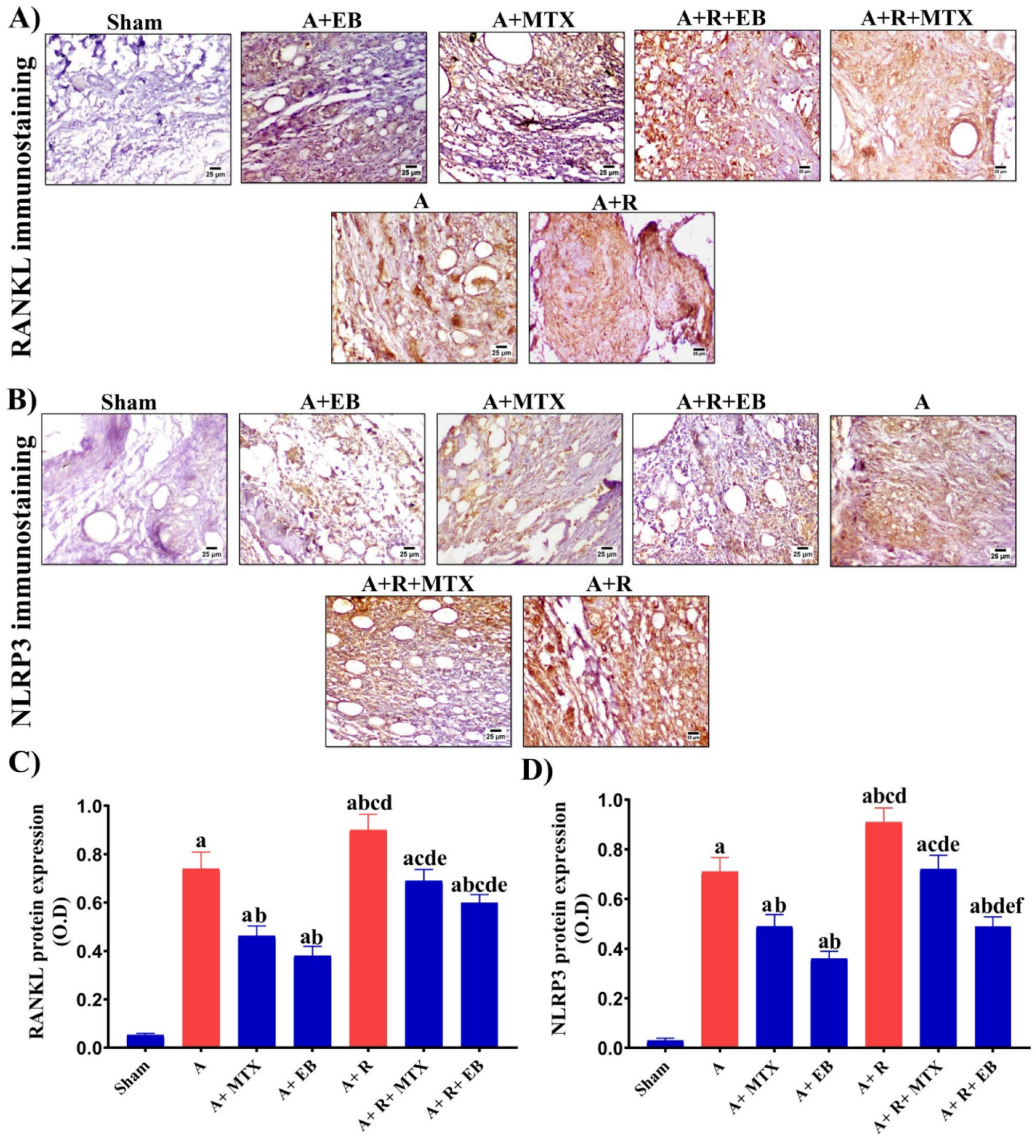
EB attenuates osteoclastogenesis and inflammatory cascade in the ankle joints of arthritic-irradiated rats through hindering RANKL and NLRP3 expression**.** **A** Representative photomicrographs indicating the immunoreactivity of RANKL protein expression in the ankle joints of different groups (magnification X 400). The target protein expression is visualized as dark brown areas. **B** Representative photomicrographs demonstrating the immunoreactivity of NLRP3 protein expression in the ankle joints of different groups (magnification X 400). **C**, **D** RANKL and NLRP3 protein expression was quantified as optical density (OD) throughout at least six different fields for each rat section. Each value represents Mean ± SEM (*n* = 6–8). Columns denoted with ״a״ significant from Sham, ״b״: significant from A, ״c״ significant from A + MTX, ״d״ significant from A + EB, ״e״ significant from A + R and ״f״ significant from A + R + MTX, at (*P* < 0.05). Sham: normal rats, A: rats injected with CFA, A + MTX: rats injected with CFA and treated with MTX, A + EB: rats injected with CFA and treated with EB, A + R: rats injected with CFA and exposed to2 Gy/week; up to a cumulative dose of 6 Gy, A + R + MTX: rats injected with CFA, and exposed to 2 Gy/week; up to a cumulative dose of 6 Gy and treated with MTX, and A + R + EB: rats injected with CFA, and exposed to 2 Gy/week; up to a cumulative dose of 6 Gy and treated with EB (color figure online)

We further sought to determine the expression pattern of RANKL and NLRP3 proteins in the ankle joints of the arthritic and arthritic irradiated rats, whether treated or untreated, to highlight the mitigative potential of the investigated treatments. FCA inoculation to rats caused a considerable upregulation (*P* ˂ 0.05) in RANKL and NLRP3 expression by 14.2- and 23.6-folds, respectively, as shown in the A group versus the sham animals, as illustrated by Fig. 5A–D. Oppositely, when compared to the A group, MTX markedly diminished (*P* ˂ 0.05) RANKL and NLRP3 expression by 39.2% and 32.5%, respectively, as observed in the A + MTX group. Surprisingly, a comparable effect was noticed in the A + EB group, but to a greater extent, as EB administration to arthritic rats proficiently reduced (*P* ˂ 0.05) RANKL and NLRP3 expression by 48.6% and 49.2%, respectively, when compared to the A group (Fig. 5 A-D). Whereas, exposure of arthritic rats to fractionated whole body γ-radiation (A + R group) boosted (*P* ˂ 0.05) RANKL and NLRP3 expression by 1.22- and 1.3-folds, respectively, when compared to the A group (Fig. 5 A-D). However, in the A + R + MTX group, there was a significant abolishment (*P* ˂ 0.05) in RANKL and NLRP3 expression by 23.3% and 21%, respectively, when compared to the A + R group. Most importantly, administration of EB to arthritic irradiated rats in the A + R + EB group effectively curtailed (*P* ˂ 0.05) RANKL and NLRP3 expression by 33.3% and 46%, respectively, when compared to the A + R group (Fig. 5A–D).

### EB maintains integrity and prevents apoptosis in the joints’ cartilages of arthritic-irradiated rats

**Fig. 6:**
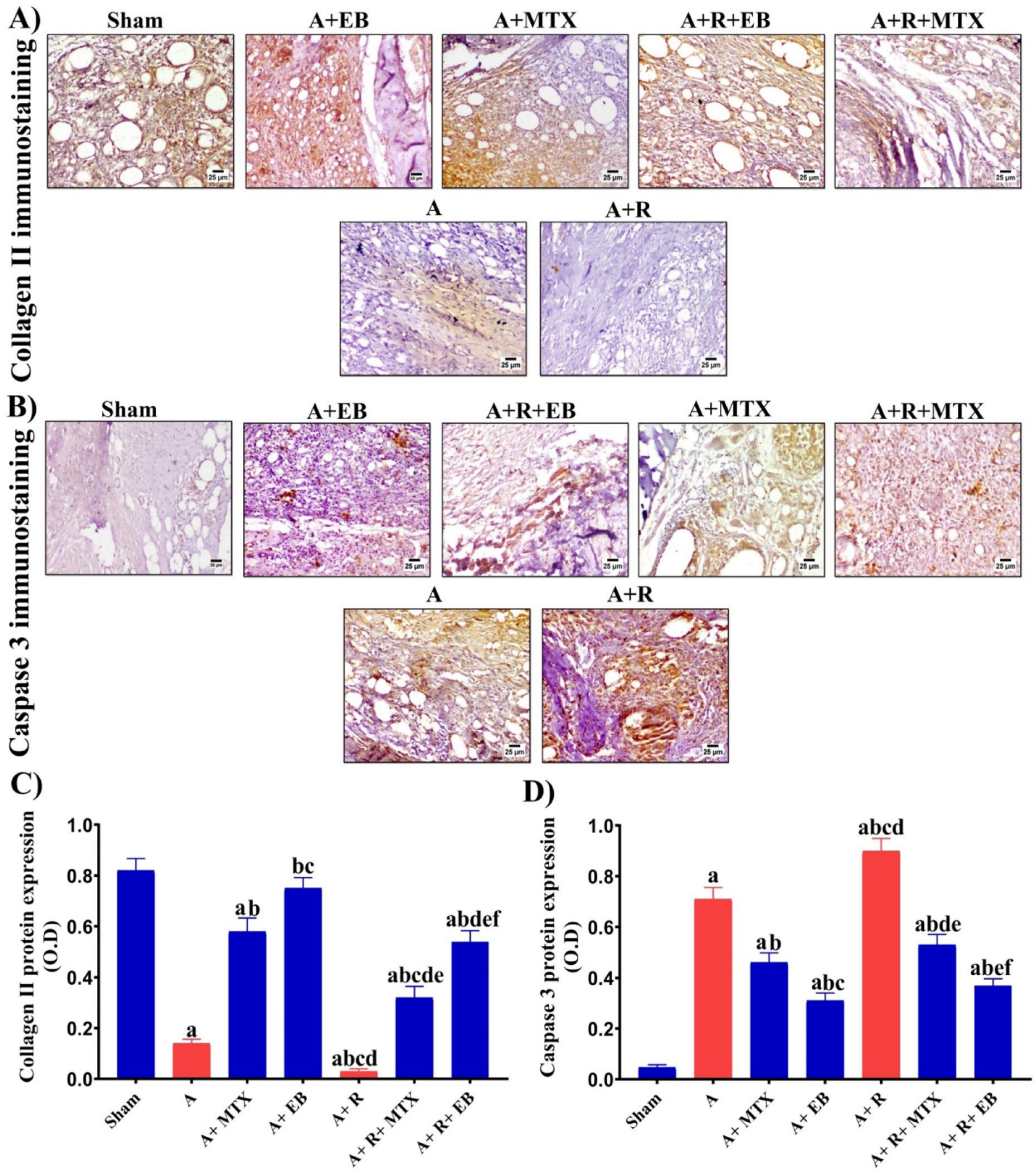
EB restores collagen type II and suppresses caspase-3 protein expression in arthritic-irradiated rats. **A** Representative photomicrographs revealing the immunoreactivity of collagen type II protein expression in the ankle joints of different groups (magnification X 400). The target protein expression is visualized as dark brown areas. **B** Representative photomicrographs demonstrating the immunoreactivity of caspase-3 protein expression in the ankle joints of different groups (magnification X 400). **C**, **D** Collagen type II and caspase-3 protein expression was quantified as optical density (OD) throughout at least six different fields for each rat section. Each value represents Mean ± SEM (*n* = 6). Columns denoted with ״a״ significant from Sham, ״b״: significant from A, ״c״ significant from A + MTX, ״d״ significant from A + EB, ״e״ significant from A + R and ״f״ significant from A + R + MTX, at (*P* < 0.05). Sham: normal rats, A: rats injected with CFA, A + MTX: rats injected with CFA and treated with MTX, A + EB: rats injected with CFA and treated with EB, A + R: rats injected with CFA and exposed to2 Gy/week; up to a cumulative dose of 6 Gy, A + R + MTX: rats injected with CFA, and exposed to 2 Gy/week; up to a cumulative dose of 6 Gy and treated with MTX, and A + R + EB: rats injected with CFA, and exposed to 2 Gy/week; up to a cumulative dose of 6 Gy and treated with EB (color figure online)

FCA inoculation to rats induced a significant reduction (*P* ˂ 0.05) in collagen type II protein expression by 83% as well as substantially raised (*P* ˂ 0.05) caspase-3 by 15-fold, as noticed in the A group when compared to the sham set (Fig. 6A–D). Obviously, MTX and EB were proficiently capable of maintaining the structural integrity of the joints in arthritic rats, as indicated by markedly restoring (*P* ˂ 0.05) collagen type II protein expression by 4- and 5.3-folds, and significantly suppressing (*P* ˂ 0.05) caspase-3 protein expression by 35% and 56%, respectively, as observed in the A + MTX and A + EB groups when compared to the A group. In contrast, exposure of arthritic rats to fractionated whole body γ-radiation (A + R group) significantly limited (*P* ˂ 0.05) collagen type II protein expression by 96.3%, while inducing a remarkable enhancement (*P* ˂ 0.05) in caspase-3 protein expression by 21%, when compared to the A group (Fig. 6A–D). Noteworthy, treatment of arthritic irradiated rats with MTX or EB as indicated in A + R + MTX and A + R + EB groups significantly replenished (*P* ˂ 0.05) collagen type II protein expression by 10.6- and 18-folds, while profoundly suppressing (*P* ˂ 0.05) caspase-3 protein expression by 41% and 58%, respectively, when compared to the A + R group (Fig. 6A–D).

## Discussion

In terms of inflammatory cell infiltration, synovial hyperplasia, synovitis, and cartilage degradation, Freund's complete adjuvant (FCA)-induced RA in rats is a reproducible, authoritative, and substantiated model with a reasonable experimental duration and clinical manifestations resembling RA in human. Thus, it is commonly conducted in pharmacological screening of anti-arthritic drugs in the preclinical stage (Jia et al. 2016; Guo et al. 2018a). As the disease progresses, the synovial membrane, cartilage, and bone are gradually destroyed, eventually leading to joint malformation (Nogueira et al. 2016). In this study, inoculation of FCA into rats' hind paws led to the development of a classic AIA in vivo model, characterized by steadily exacerbated arthritis-related manifestations, whereby a paw swelling, body weight gain, joint histopathological debilitating lesions, clinical arthritic score, global arthritic assessment scoring, and macroscopic observation of paw edema were employed to determine the degree of arthritis and the mitigative potential of EB and MTX. The body weight of A and A + MTX groups dropped drastically, whereas the body weight of sham and A + EB groups was increased gradually. In FCA-induced arthritic rats, the magnitude of systemic inflammation is tightly associated with body weight (Ahmed et al. 2019). The loss of body weight in the A group rats could be due to an increase in leptin production caused by FCA challenge, which may then contribute to a decrease in food intake, a lack of appetite, and ultimately weight loss (Kadhem 2016). Loss of weight in arthritic rats is primarily attributed to prolonged joint inflammation caused by local or general implications of pro-inflammatory cytokines generated by monocytes and macrophages (Choy and Panayi 2001), which could elicit muscle degeneration (Shokry et al. 2022). As reported earlier, MTX treatment could enhance the cytotoxic effects in AIA animals, allowing for additional weight loss in synergy with the detrimental aspects in FCA arthritic rats (Hasan et al. 2018). In agreement with previous research, our findings revealed prevalent macroscopic and microscopic indicators of arthritis (Asenso et al. 2019; Sun et al. 2021; Shokry et al. 2022). TNF-α and IL-1β, as well as other pro-inflammatory cytokines produced by activated macrophages and synovial fibroblasts are major inflammatory factors in RA pathogenesis (Bakhtiari et al. 2019; Dong et al. 2020a, b). Moreover, NLRP3-mediated caspase-1 activation plays a crucial role in osteoarthritis and RA progression **(**Guo et al. 2018b; Zu et al. 2019). These pro-inflammatory cytokines are found not only in joints and synovial fluids but also in serum (Chiang et al. 2019), which supports their overexpression in serum and synovial tissue in the current study and explains the systemic inflammatory characteristics of RA. In RA, TNF-α stimulates the cytokine cascade by increasing pro-inflammatory cytokines while hindering anti-inflammatory cytokines such as IL-4 and IL-10 (Choy and Panayi 2001). TNF-α and IL-1β, two pro-inflammatory cytokines, are presumed to be key determinants to long-term synovitis, synovial hyperplasia, and, eventually, cartilage and bone obliteration (Chen et al. 2019). IL-1β and its upstream mediator, TNF-α, play pivotal roles in the immunological and inflammatory responses in RA development, activating leukocytes, endothelial cells, synovial fibroblasts, and osteoclasts, triggering the production of adhesion molecules and matrix enzymes, boosting inflammatory cytokine signaling pathways, and obstructing regulatory T-cell function (Zampeli et al. 2015). The elevated levels of these pro-inflammatory cytokines in synovium may play a vital role in the joint injury and debilitating lesions noted in the microscopic sections examined in our study.

The emergence of oxidative stress or redox disparity is triggered by an excess of the various reactive oxygen species (ROS), whether through increased production, a reduction in antioxidant defenses, or a blend of the both. Oxidative stress is essential in the development of RA (da Fonseca et al. 2019). Actually, oxidative stress is related to clinical features of symptom severity in RA (Balogh et al. 2018). Furthermore, minimal concentrations of antioxidant defenses have been indicated in RA patients' serum and synovial fluid (Oztürk et al. 1999). Therefore, numerous investigations have found a switch in the oxidant/antioxidant equilibrium endorsing the former in RA serum, synovial tissue, and fluid, leading to the appearance of oxidative damage in cartilage (Balogh et al. 2018; Pradhan et al. 2019; Alcaraz and Ferrándiz 2020). Elevated cytokine production induces inflammatory cells such as neutrophils and macrophages to release ROS into synovial fluid, thereby facilitating tissue injury (Wang et al. 2022). In the current study, FCA immunization induced arthritis produced a considerable increment in oxidative stress biomarkers, as evident by elevated levels of serum and synovial ROS and MDA levels, along with a notable abolishment of SOD, CAT, GPx, and GSH levels in serum and synovial tissue of the A group, which is in agreement with previous studies (Al-Muhtaseb et al. 2019; Shabaan et al. 2022). In addition, RANKL, NLRP3, and caspase-3 protein expression showed an overexpression, paralleled by a significant reduction in collagen type II expression in the ankle joints of the A group, as seen in our study. The current findings are in accordance with the observations of previous studies (Wang et al. 2020; Jing et al. 2021; Abdel-Rafei et al. 2022). The accumulation of pro-inflammatory cytokines in the synovium of RA patients promotes the expression of RANKL, which is required for osteoclast differentiation (Chang et al. 2016). Osteoclasts are largely responsible for joint bone deterioration in RA. Osteoclasts are participants of the monocyte/macrophage lineage and are the only cells involved in bone resorption by governing anabolic and catabolic processes of the osseous tissue (Ren et al. 2022). During osteoclastogenesis, RANKL-induced RANK activation generates ROS, which further activates the RANKL-mediated signaling cascade (Kim et al. 2017). Moreover, caspase-1 activation and IL-1β secretion result from NLRP3 inflammasome assembly (Yang et al. 2019). ROS are signaling intermediates that can induce both the NLRP3 inflammasome and the NF-κB (Zhao et al. 2019). Even though controlled apoptotic death retains cartilage homeostasis (Caramés et al. 2015), exaggerated apoptosis caused by the local inflammatory environment poses a major obstacle in OA treatment (Dai et al. 2018). It has been proven that IL-1β can cause mitochondrial dysfunction-related apoptosis in chondrocytes (Wang et al. 2021). Apoptosis performs a crucial role in the development of arthritic pathologies. Accelerated rates of apoptosis hamper chondrocyte survival and function (Hwang and Kim 2015).

The present study found that exposing arthritic rats to fractionated whole body γ-irradiation (2 Gy/fraction for 3 successive weeks; A + R group) led to marked worsening in the clinical arthritic signs, biochemical indices of oxidative stress and inflammation in serum and synovial tissue, and degenerative lesions in the ankle joints. There is ample evidence supporting the therapeutic potential of fractionated low dose radiation (LDR) in arthritic disorders (Deloch et al. 2018; Donaubauer et al. 2020; El-Ghazaly et al. 2020; Abdel-Rafei et al. 2022). However, the influence of high dose radiation (HDR) delivered in fractions (~ 2 Gy/ fraction) on the severity of arthritic signs is not well understood. The processes of bone metabolism, particularly osteoclasts (OCs) and osteoblasts (OBs) differentiation and functionality, are strictly controlled. As a result, those mechanisms are vulnerable to a variety of interruptive factors. Internal factors such as menopausal hormonal imbalances or rheumatic disorders can produce catastrophic skeletal alterations (Almeida et al. 2017). External influences, on the other hand, can impair the bone in general, together with OCs and OBs. Therapeutic medications, mechanical stress, and ecologic or medically delivered ionizing radiation (IR) are among these factors (Shanmugarajan et al. 2017). Radiotherapy (RT) has become the most commonly utilized treatment for cancer, with over 60% of patients with solid tumors receiving it (Orth et al. 2014). Through generation of ROS, the "target effect" of radiation causes DNA damage in cells, that becomes mainly accountable for cancer progression regulation (Balagamwala et al. 2013). Nevertheless, it has become broadly recognized that RT could also elicit anticancer effects through boosting the immune system, the well-known "non-targeted or abscopal effect," although the mechanisms by which radiation activates the immune system (i.e., total dose, daily dose, and timing) are not fully understood (Deloch et al. 2016). The ability of RT to augment antigen presentation is among the proposed approaches associated with the non-targeted effect (Kamrava et al. 2009; Fiorica et al. 2021). Based on this, an exaggerated immune response could be related to the reinforcement and/or exacerbation of symptoms in patients with autoimmune conditions undertaking RT (Fiorica et al. 2021). Antigen-presenting cells (APCs) are essential in triggering and/or maintaining the chronic inflammatory process in RA (Rodríguez-Fernández 2013). Different studies have reported that IR has a deleterious impact on OB functionality, which includes collagen production, as well as OB proliferation. Besides, IR has been shown to cause cell cycle arrest in OBs (Sakurai et al. 2007). Because high doses of IR produce a damage response and inflammatory cascades in exposed tissues like bone, pro-inflammatory cytokines like TNF-α, IL-6, and IL-1β may be secreted. In such an inflammatory environment, OC differentiation can be enhanced because TNF-α and IL-1β directly activate RANKL expression (Willey et al. 2011). Moreover, NLRP3 activation was found to mediate radiation- induced multiple organ damage (Liu et al. 2017; Wei et al. 2019). Accordingly, all these factors and mechanisms might contribute immensely to aggravated severity of the arthritic signs observed in the present study.

According to the findings of the current investigation, EB administration to either arthritic (A + EB group) or arthritic irradiated (A + R + EB group) rats displayed a notable improvement in the FCA-induced degenerative changes in the ankle joint. Generally, this could be attributed to its antioxidant and anti-inflammatory properties. In Particular, it was shown that the administration of EB restored the antioxidant enzymes activities (SOD, CAT, and GPX) as well as GSH levels associated with decreased lipid peroxidation in a rat model of cisplatin-induced nephrotoxicity (Husain et al. 1998). Also, EB attenuated ischemia reperfusion-induced cardiomyocyte apoptosis through decreasing the expression of caspase-3 and enhancing the expression and function of antioxidant enzymes (Cheng et al. 2019), in addition to its peripheral antioxidant effect and its effect on the reversal of renal lipid peroxidation, as demonstrated in the study of Klann et al. (2020) against oxidative stress induced by a model of Alzheimer's disease. The study by Kushwah et al. (2015) found that the addition of EB to cultured lung epithelial cells results in a reduction in ROS production, lipid peroxidation, and cytokine production. Furthermore, the study of Groß et al. (2016) suggests that ROS-induced NLRP3 activation can be counteracted by thiol-active antioxidants as peroxidase mimetic ebselen. Moreover, Hamarsheh et al. (2020) found that blocking of ROS generation by EB abrogated the elevation of caspase-1 cleavage and subsequently reduced IL-1β production in bone marrow derived dendritic cells of Kras^G12D^ knock-in mice. In addition, EB treatment was found to suppress the TNF-α induced pro-inflammatory stimulators in glioblastoma, preventing the accumulation of a detrimental effect of pro-inflammatory mediators in the microenvironment (Tewari et al. 2009). Several studies have shown that in a murine microbial infection models, treatment with EB significantly reduced the microbial load as well as its consequences on the elevation of pro-inflammatory cytokine expression such as TNF-α, IL-6, and IL-1β (Dong et al. 2020a, b; Sakita et al. 2021). Additionally, the study of Thabet and Moustafa (2017) demonstrated that EB reduces the protein expression of NF-κB and increases the expression of the anti-inflammatory cytokine IL-10, which agrees precisely with the current findings. Besides, it was found in the study of Chew et al. (2010) that EB caused a significant decline in collagen-I levels due to its GPx-mimic properties in a diabetic model associated with GPx deficiency. Collagen type I was found to be significantly implicated in the pathogenesis of arthritic diseases, such as osteoarthritis and RA (Miosge et al. 2004). As a result, EB prevents the preponderance of denatured collagen type I while maintaining the native collagen type II expression levels, affording protection against RA progression.

## Conclusion

**Fig. 7:**
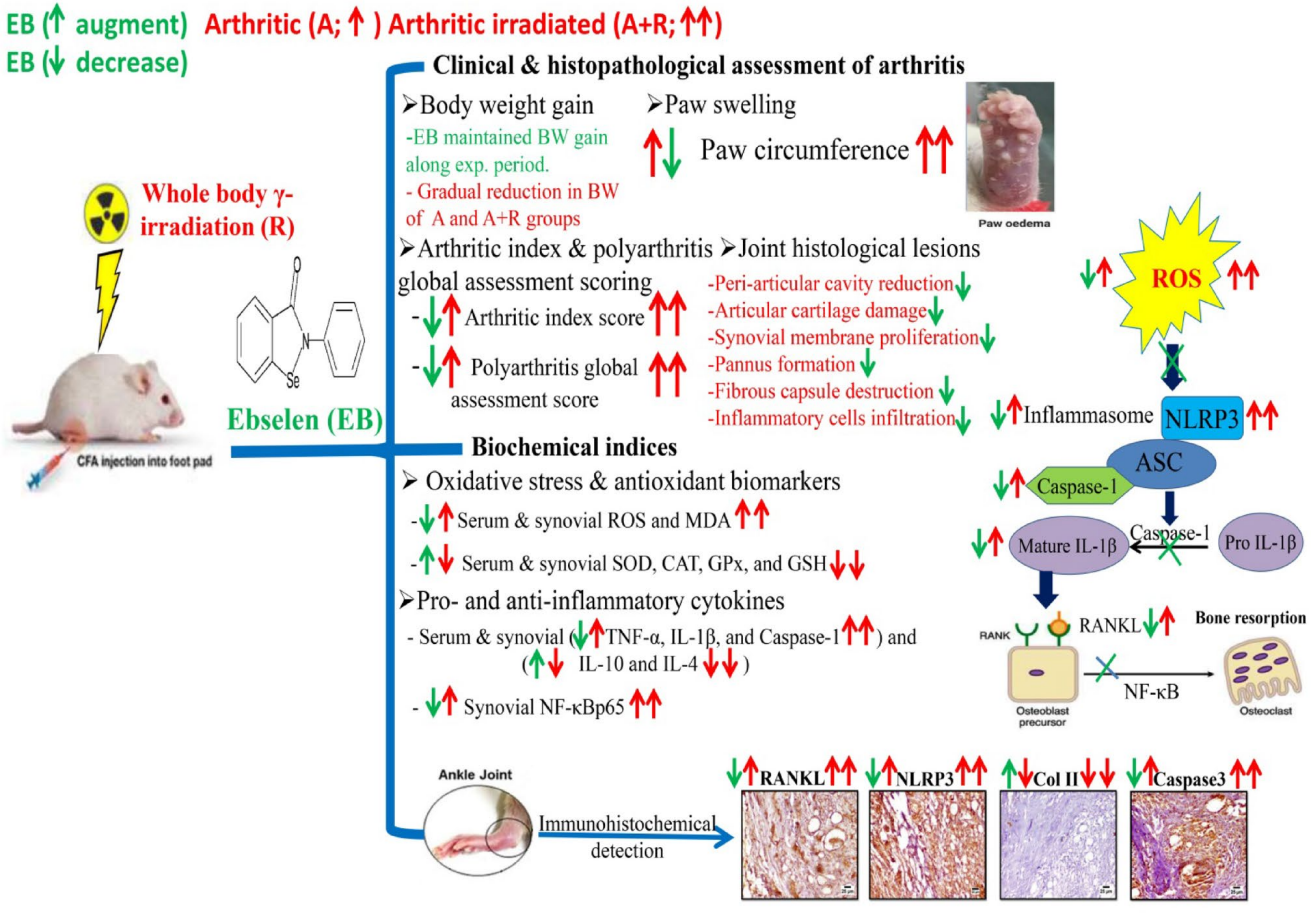
The mode of action involved in the anti-arthritic potential of ebselen (EB) against the degenerative lesions induced by Freund's complete adjuvant (FCA; A) and exposure to whole body γ-irradiation (A + R) in the joints of rats is depicted schematically

Through its antioxidant and anti-inflammatory properties, it is possible to deduce that EB has anti-arthritic and radioprotective effects and improves the severity as well as the joint degenerative lesions in arthritic and arthritic-irradiated rats. These effects could be attributed to the ability of EB to scavenge ROS, improve the antioxidant system, reduce lipid peroxidation in serum and synovial tissue, and restrain NLRP3 activation, which then prevents caspase-1/IL-1β/RANKL/NF-κB pathway induction, as demonstrated by its effect on diminishing TNF-α, caspase-3, and retrieval of collagen-II expression in ankle joints, while enhancing anti-inflammatory cytokine expression (IL-4 and IL-10) in serum and synovial tissue (As shown in Fig. 7).

## Data Availability

The generated and analyzed data that support the findings of this study are included within the manuscript.
